# The Effect of Chemical and Physical Treatment on Morphology, Wet Strength, and Water Absorption of Kapok Fiber-Based Absorbent

**DOI:** 10.1021/acsomega.6c05757

**Published:** 2026-07-30

**Authors:** Nur Syazwani Abd Rahman, Mohd Firdaus Yhaya, Abdul Fattah Nongman, Baharin Azahari, Wan Ruslan Ismail, Norhafizah Saari

**Affiliations:** † Bioresource Technology, School of Industrial Technology, Universiti Sains Malaysia, 11800 Penang, Malaysia; ‡ Biomaterials Synthesis Laboratory (BSL), School of Dental Sciences, Universiti Sains Malaysia, 16150 Kubang Kerian, Kelantan, Malaysia; § Section of Geography, School of Humanities, Universiti Sains Malaysia, 11800 Penang, Malaysia; ∥ Department of Microbiology and Parasitology, School of Medical Sciences, Health Campus, Universiti Sains Malaysia, 16150 Kubang Kerian, Kelantan, Malaysia

## Abstract

Paper-based absorbents have attracted increasing attention as sustainable fiber-based materials for wet-environment applications. However, their limited wet strength remains a major challenge, particularly when the absorbent is exposed to water or moisture during use. Therefore, this study focused on improving the wet strength of kapok fiber-based handsheet absorbents for potential use under wet conditions. Sheet adsorbents made of fibrillated kapok fibers of different lengths were prepared by exposing the kapok fibers to pretreatment using sodium hypochlorite (TT), followed by the wet ball milling (BM) technique for a certain duration. Azide–alkyne click modification was employed to enhance the mechanical properties of the handsheet-based adsorbent under wet conditions. On the basis of the wet strength study, the clicked handsheet performed better in both wet tensile and wet bursting properties as compared with the control condition. The clicked TT1 BM6 handsheet was the best as far as wet strength and water absorption were concerned. The enhancement of wet strength shows the great potential of the clicked kapok fibers to be utilized as adsorbents under wet conditions.

## Graphical abstract

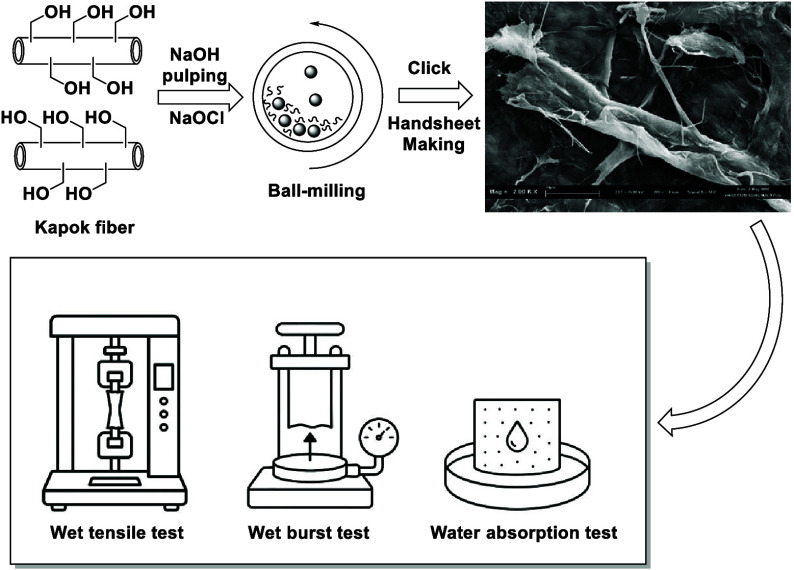

## 1 Introduction

Paper-based absorbents have gained increasing attention as sustainable, lightweight, and porous fiber-based materials for water-related applications. Paper-based absorbents recently have been introduced as an alternative for synthetic polymer membranes.^1–4^ Nanopaper has become one of the famous alternatives compared with synthetic membrane polymers in the ultrafiltration process. The fabrication of paper-based absorbents follows the basic principles of papermaking, in which fiber morphology, surface characteristics, and fiber-to-fiber bonding strongly influence the final structure and performance of the sheet. Kapok fibers were selected as the raw material in this study due to their high internal porosity of more than 80% by volume compared with other natural fibers.^13,14^ This highly porous structure is useful for increasing the surface area available for chemical modification and absorbent-related applications.

In paper-based absorbents, fiber morphology plays an important role in determining the sheet structure and mechanical performance. Fiber surface area affects the number of available contact sites between fibers, which can influence the absorbent performance of the sheet.^5^ Fiber length also affects the mechanical behavior of paper, as shorter fibers may reduce fiber bridging, entanglement, and load transfer, whereas excessively long fibers may curl and form a less uniform sheet structure.^6,7^ Therefore, an appropriate fiber length is required to promote effective fiber-to-fiber contact and a bonded area. Basically, the strength of the paper depends on hydrogen bonding between the fibers. Since the application of the paper-based absorbent involves direct contact with water for a certain duration, the wet strength is important.^8,9^ Ball milling was applied to fibrillate the kapok fibers and increase the available contact area for fiber-to-fiber bonding. However, hydrogen bonding alone may not provide sufficient strength to the paper, especially when the paper is exposed to water. Therefore, a chemical cross-linking strategy that can provide more stable interfiber bonding is needed to improve the wet strength of kapok fiber-based handsheet absorbents.

In this study, azide–alkyne click chemistry was introduced as a chemical cross-linking strategy to improve the wet strength of kapok fiber-based handsheet absorbents. To increase the wet strength of the handsheet, azide–alkyne click chemistry was introduced to join these fibers permanently.^10,11^ Some parts of the fiber were treated using sodium azide to produce azidated fiber, while the other parts of the fiber were treated with propargyl bromide to produce propargylated fiber. Since click chemistry is very selective, the reaction will only take place between the azide and alkyne components, forming a triazole ring.^12^ Herein, handsheets were fabricated from kapok fibers with different sizes, produced by sodium hypochlorite pretreatment and wet ball milling. The ball-milled fibers were then functionalized into azidated and propargylated fibers before being clicked through azide–alkyne reaction to form cross-linked handsheets. The effects of chemical treatment, ball milling, and click modification on fiber morphology, wet tensile strength, wet bursting strength, porosity, and water absorption were evaluated to determine the most suitable kapok fiber-based handsheet absorbent for wet-condition applications.

## 2 Experimental Section

### 2.1 Pretreatment of Kapok Fibers

Soda pulping with some modifications was done on the raw kapok fibers to remove wax, hemicellulose, extractives, and impurities (Guo et al.).^11^ The removal of wax from inside the lumen would create more hollow fibers, increasing the surface area for the consequent chemical modifications and adsorption. 300 g of oven-dried kapok fibers was mixed with 825 mL of 2 M NaOH (25% active alkaline charge). Distilled water was added to achieve a liquid-to-fiber ratio of 7:1 in a 20 L static digester. 0.01% Anthraquinone based on the oven-dried weight of fiber was added. The pulping process was done at 170 °C. The time taken to reach 170 °C was 90 min, before holding up at 170 °C for another 120 min. Once done, the newly formed pulp was then rinsed with water, before being refined by a refiner at a plate gap of 70° to shorten the fiber. The cleaned fiber was screened using a screening machine to remove undesirable fibrous and nonfibrous materials, before oven drying at 80 °C until a constant weight was reached.

### 2.2 Fiber Treatment with Sodium Hypochlorite and Wet Ball Milling Process

10 g (weight based on oven-dried) of pretreated kapok fibers was soaked in 10% sodium hypochlorite with a ratio of 1:10 (dry weight fiber:sodium hypochlorite) to remove lignin. The fibers were soaked for 1, 3, and 5 days at room temperature before washing with distilled water. Then, the treated fibers were ball-milled at room temperature at 1200 rpm with different milling times. The milled fibers were then stored in a plastic bottle before further experiments.

### 2.3 Preparation of Tosylated Fibers

4 g of ball-milled fibers was mixed with 9.44 g of para-toluenesulfonyl chloride (tosyl chloride, TsCl), 11.84 mL of triethylamine (TEA), and 160 mL of distilled water under mechanical stirring for 24 h at room temperature. The mixtures were then filtered and washed with 300 mL of hot distilled water, followed by 300 mL of hot ethanol to remove the unreacted tosyl chloride, before drying at 40 °C until constant weight was achieved.

### 2.4 Azidation of Tosylated Fibers

The azidation process was carried out using the tosylated fibers. 2 g of tosylated fibers was mixed with 3.85 g of sodium azide in 60 mL of *N*,*N*-dimethylformamide (DMF) under stirring at 75 °C. The reaction was conducted for 48 h, and the mixture was cooled down to room temperature, followed by filtration using filter paper. The product was washed using hot distilled water and hot ethanol as described in the previous method, before drying at 40 °C until constant weight was achieved.

### 2.5 Preparation of Propargylated Fibers

1.0 g (based on oven-dry weight) of ball-milled fibers was mixed with 30 mL of distilled water. Then, 12 mL of propargyl bromide was added dropwise into the mixture. The stirring and the reaction were carried out for 24 h at room temperature. The mixture was then filtered and washed with distilled water and ethanol to remove excess and unreacted propargyl bromide. The propargylated fiber was dried at 40 °C overnight until constant weight was achieved.

### 2.6 Clicking of Fibers

The click reaction was carried out by mixing 0.5 g of azidated fibers and 0.5 g of propargylated fibers in 75 mL of DMF. The mixture was stirred and left to react at 100 °C for 24 h. The resulting mixture was then filtered and washed with hot distilled water and hot ethanol to remove the DMF. Then, the clicked fibers were ready for the handsheet making process.

### 2.7 Handsheet Making Process

First, the pulp stock was prepared by adding 10 g (based on dry weight) of clicked fibers into 1500 mL of distilled water. Then, the consistency of the pulp stock was adjusted to 0.3% by adding the appropriate amount of distilled water. Then, the clicked handsheet was produced according to Lee and co-workers and TAPPI Method T-205sp-02 with some modifications. The targeted basis grammage of the handsheet was 40 g/m^2^ to obtain a constant weight of fiber for each handsheet. The resulting handsheet was allowed to dry completely for 24 h before further processing.

### 2.8 Fiber Length Measurement

The length of the ball-milled fiber was determined by using a fiber length analyzer (Malvern Master). About 2 mL of the fiber dispersion sample was poured into a measuring chamber. The fiber dispersion sample was sucked and passed through the optical measuring instrument. The fiber length of the sample was measured by using a fractionator. The weight-average fiber length was estimated.

### 2.9 Fourier Transform Infrared (FTIR)

The FTIR analysis was conducted using an FTIR spectrometer (Nicolet iS10, Thermo Scientific) to determine and analyze the effect of modification on the functional groups of the modified kapok fibers. The FTIR spectra were recorded over the wavenumber region of 4000–400 cm^–1^. Approximately 5 mg of sample (raw kapok fiber, pretreated, and modified kapok fiber) was mixed with 95 mg of fine-ground potassium bromide (KBr) and pressed to form a transparent pellet. Then, the pellet was inserted into a disc holder and placed in the spectrometer, followed by scanning. All of the modified kapok fibers, such as tosylated, azidated, propargylated, and clicked kapok fibers, were compared with the control kapok fibers.

### 2.10 Field Emission Scanning Electron Microscopy (FESEM)

The surface morphology of the untreated and treated samples was observed using field emission scanning microscopy (Model LEO Supra 50 Vp). The samples were cut using a gold cutter and glued on the aluminum stub and coated with a thin layer of gold using a sputter coater (model Polaron DC 515 ± 20 nm) to avoid charging. The sample images were viewed and captured at different magnifications.

### 2.11 Wet Tensile Strength (Tensile Index)

The wet tensile strength of the handsheet adsorbent was determined using a FRANK-PTI horizontal machine according to TAPPI Standard T 494. The handsheet adsorbent samples with dimensions 210 mm × 25 mm were prepared. Before the testing was conducted, the sample was soaked in 100 mL of distilled water until it was totally wet. Then, excess water was removed by using a roller and blotting paper. Then, the samples were placed on the plane of the machine and clamped on both sides with an initial test span of 90 mm. The testing was set with a 500 N load and a uniform rate of 25 mm ± 5 mm/min. The wet tensile index was calculated based on eq 1. Five replicate samples were conducted, and the average result was recorded.

1 $$\mathrm{wet}\;\mathrm{tensile}\;\mathrm{index}\;\left(\frac{\mathrm{Nm}}{g}\right)=\frac{\mathrm{wet}\;\mathrm{tensile}\;\mathrm{strength}\;\left(\frac{N}{m}\right)}{\mathrm{grammage}\;\left(\frac{g}{m2}\right)}\times 100$$

### 2.12 Wet Bursting Strength (Bursting Index)

Wet bursting strength of the handsheet adsorbent samples was conducted using a Mullon Burst Tester according to TAPPI 403 om-91. Before the testing was conducted, the sample was soaked in 100 mL of distilled water until totally wet. Then, excess water was removed by using a roller and blotting paper. Then, the sample was placed at the clamping area and hydrostatic pressure at a rate of 95 ± 5 mL/min was applied through a rubber diaphragm. The maximum hydrostatic pressure required to produce rupture of the handsheet was recorded. The bursting index was calculated based on eq 2.

2 $$wet\;bursting\;index\;\left(kPa\cdot \frac{m2}{g}\right)=\frac{wet\;bursting\;strength\;\left(kPa\right)}{grammage\;\left(\frac{g}{m2}\right)}$$

### 2.13 Water Absorption

Water absorption was used to measure the water uptake and water holding capacities until the saturation point of the handsheet adsorbent. The testing was conducted according to Liu et al. with some modifications. Samples with 2.54 cm × 2.54 cm dimensions were weighed in its dry form, immersed in 50 mL of distilled water, and the weight of the sample was taken at the interval time up to 48 h. Upon reaching the interval time, the excess water at the surface of the sample was removed using a blotting paper before the sample was weighed. The percentage of water absorption of the sample was calculated as eq 3

3 $$\mathrm{water}\;\mathrm{absorption}\;\left(\%\right)=\frac{W2-W1}{W1}\times 100$$

where *W*1 is the initial weight of the sample and *W*2 is the final weight of the sample for each measurement.

### 2.14 Porosity

The porometer Porolux 1000 series was used to measure the porosity of the handsheet adsorbent. The pore size and pore distribution were characterized by a gas–liquid displacement method where the handsheet samples were prewetted using a commercial wetting liquid and nitrogen gas was used to push the liquid out of the membrane pores. The pressure and permeation flow of the nitrogen gas through the opened pores were recorded (Wang et al.).

## 3 Results and Discussion

### 3.1 Fourier Transform Infrared (FTIR) Analysis

The FTIR analysis was used to analyze the effect of chemical modifications on the kapok fibers (Figure 1). The chemical modification of kapok fibers involved sequential dewaxing, tosylation, azidation, or propargylation, followed by an azide–alkyne click reaction between the azidated and propargylated fibers to form clicked kapok fibers.

**Figure 1:**
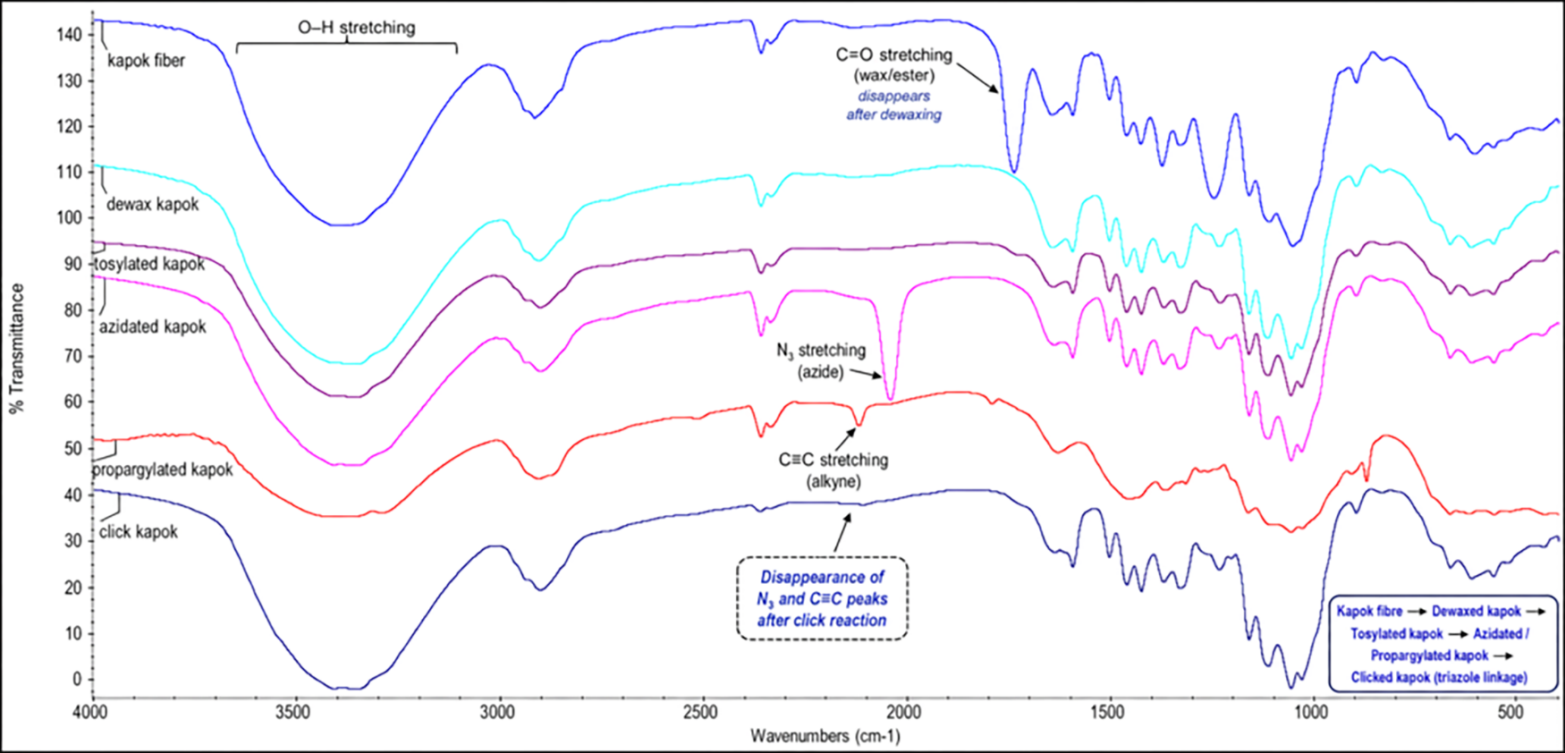
FTIR spectra of raw kapok fiber, dewaxed kapok fiber, tosylated kapok fiber, azidated kapok fiber, propargylated kapok fiber, and clicked kapok fiber, with labeled characteristic peaks indicating the surface chemical changes after modification.

The successful dewaxing of kapok fibers was proven by the total disappearance of the peak at 1700 cm^–1^ related to the presence of carbonyl (–C=O) group stretching of ester, aliphatic aldehydes, esters, and ketones of plant wax. Azide–alkyne clicking involved the conjugation reaction between both azidated and propargylated fibers. However, to create the azidated fibers, the tosyl functional group must be first introduced to the dewaxed fibers, as the hydroxyl (−OH) group on fibers is a poor leaving group.^15^ The tosyl moiety acted as an excellent leaving group in nucleophilic substitution reactions and later can be substituted by azide, halogens, or an amine group.^16,17^

The −C–O–S– bonds of tosyl groups could be observed as a small peak at 1700 cm^–1^. For azidated kapok fibers, a new peak at range 2050–2100 cm^–1^ was observed, indicating the presence of the azide (–N_3_–) functional group.^18,19^ For propargylated kapok fibers, it was clearly seen that a new band at 2126 cm^–1^ was observed, corresponding to the alkyne (–C≡C−) groups.^20^ Next, after both azidated and propargylated fibers were clicked (cross-linked) together, both the peaks of azide (2050–2100 cm^–1^) and alkyne (2126 cm^–1^) had totally disappeared, confirming a successful click reaction. In the clicked kapok fibers, the triazole functional group was formed via cycloaddition.^21,22^ However, the peak at 3010 cm^–1^ related to the alkene (–C=C−) in the newly formed triazole ring was obscured inside the broad hydroxyl (−OH) peak between 3000 and 3700 cm^–1^.

### 3.2 Morphology

Longer pretreatment and ball milling times were expected to have profound effects on the morphology of the fibers, as observed by FESEM (Figures 2–6). Fibers of TT1 BM6 (midsize fibers) and TT5 BM12 (shorter fibers) were chosen to observe their differences as compared with the normal-sized (original) kapok fibers. The surface of azidated fibers (Figure 3a) was rougher and flattened as compared with the raw kapok fibers (Figure 2a) and dewaxed kapok fibers (Figure 2b). For TT1 BM6 fibers (Figure 4a), the red arrow pointed to the effect of fibrillation due to the ball milling process. The longest pretreatment (TT5) and ball milling (BM5) had the greatest fibrillation effect on the kapok fibers (Figure 5a), as the fibers were broken down into shorter ones.

**Figure 2:**
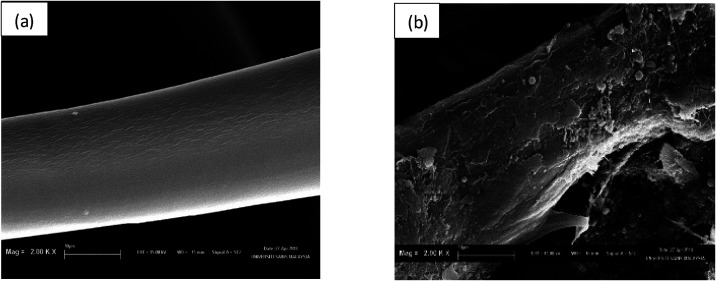
SEM images of (a) normal kapok fiber and (b) dewaxed kapok fiber.

**Figure 3:**
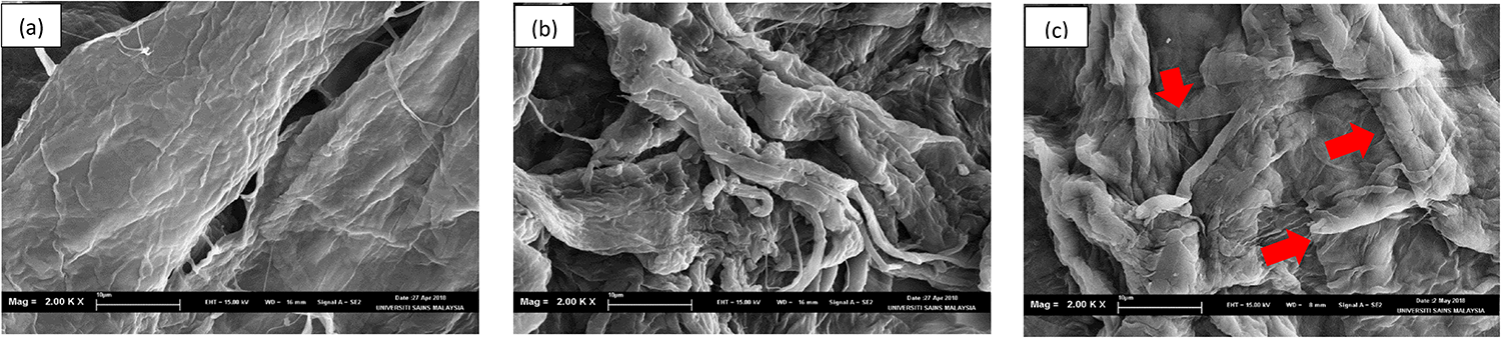
SEM images of (a) azidated fiber, (b) propargylated fiber, and (c) clicked fiber for handsheet using untreated (XTT) and unball-milled fiber (XBM).

**Figure 4:**
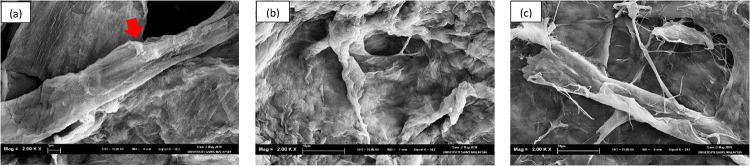
SEM images of (a) azidated fiber, (b) propargylated fiber, and (c) clicked fiber for handsheet using fiber treated with sodium hypochlorite for 1 day (TT1) and ball-milled for 6 h (BM6).

**Figure 5:**
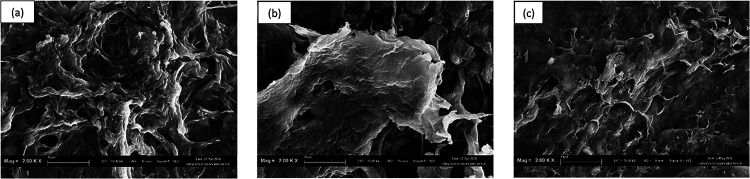
SEM images of (a) azidated fiber, (b) propargylated fiber, and (c) clicked fiber for handsheet using fiber treated with sodium hypochlorite for 5 days (TT5) and ball-milled for 12 h (BM12).

For propargylated XTT XBM fibers (Figure 3b), the effect was different compared with that of azidated fibers (Figure 3a). The fibers were rough and stringy because of cracking and disintegration behavior during fiber modification.^23,24^ A similar phenomenon was observed for TT1 BM6 (Figure 4b) and TT5 BM12 (Figure 5b) fibers. The effect of clicking (cross-linking) fibers for handsheet XTT XBM is observed in Figure 3c. The close attachment between each fiber (pointed in red arrows) confirmed the clicking process. For TT1 BM6 (Figure 4c) and TT5 BM12 (Figure 5c) handsheets, it could be seen that the attachment between the fibers became closer as the fiber length decreased.

The close attachment between the clicked fibers could also be seen on the cross-section image of the clicked handsheet, as shown in Figure 6b. For dewaxed fibers (Figure 6a), a big gap could be seen in the handsheet. After the fibers were “clicked” together, it could be seen that the gaps were closer to each other (Figure 6b). Moreover, the shorter the fiber length, the closer the space between the modified fibers, hence the reduction in the thickness of the handsheet (Figure 6c,6d). However, some of the fibrillated fibers were protruding on the surface because some of the fibrillated fibers did not fully modify during the modification process.

**Figure 6:**
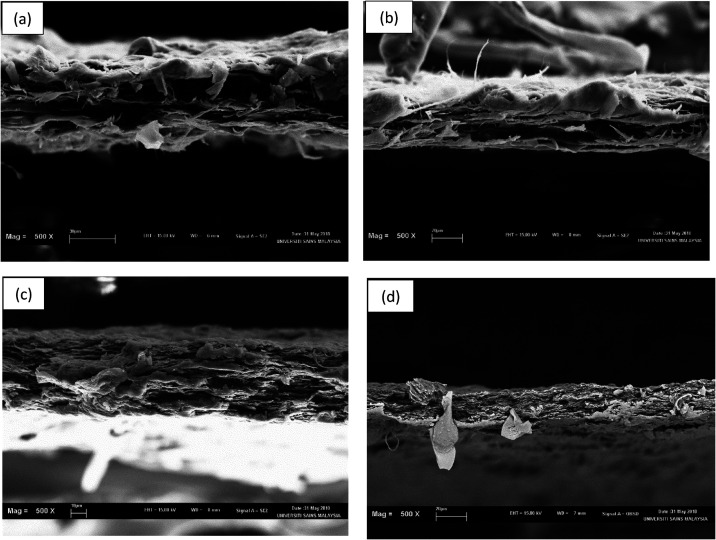
Cross-section SEM images of (a) unclicked XTT XBM (control), (b) clicked XTT XBM, (c) clicked TT1 BM6, and (d) clicked TT5 BM12 handsheet kapok fibers.

### 3.3 Wet Tensile Strength (Tensile Index)

Figure 7 shows the wet tensile index of the handsheet of clicked and nonclicked fibers with different fiber sizes. As an adsorbent, the higher the wet strength, the better. The “XTT,” “TT1,” “TT3,” and “TT5” indicated the different sodium hypochlorite treatments, which were 0, 1, 3, and 5 days, respectively. On the other hand, “XBM,” “BM1,” “BM6,” and “BM12” indicated different ball milling time conditions, which were 0, 1, 6, and 12 h milling time, respectively. The average fiber size was arranged from the smallest to the biggest size produced accordingly based on the result of the average fiber length distribution.

**Figure 7:**
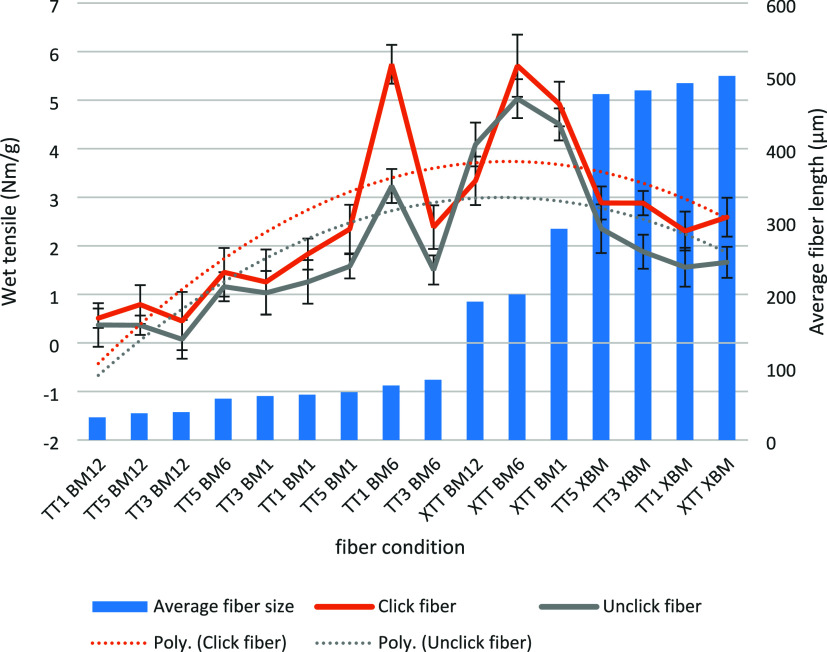
Wet tensile index for clicked and unclicked handsheets with different fiber lengths.

For both clicked and unclicked handsheets, the wet tensile indices were trending down with the decrease of fiber sizes. Most of the fibers with less than 50 μm were due to ball milling for 12 h. The long milling time had shortened the fibers. Basically, short fibers would have high surface area.^25^ However, in paper technology, the strength was more affected by the surface bounded area as compared with the overall surface area.^26,27^ As a load was applied, the stress could not be distributed evenly, causing breakage at low tensile strength. The clicked handsheet showed a much better wet tensile strength than its unclicked counterpart, as the latter solely depended on the hydrogen bonding between the fibers.^11,12^ In a dry handsheet, the hydrogen bonds hold the fibers together.

However, during contact with water, the water molecules form new hydrogen bonds by inserting themselves between the fibers, causing a rapid weakening of the fiber-to-fiber bonding.^28^ For clicked fiber, the presence of cross-linking between the alkynated fibers and azidated fibers had helped to hold them together upon exposure to water.^29^ The wet tensile for clicked handsheets was higher for TT1 BM6 (100 μm) and XTT BM6 (260 μm) handsheets. Even though the TT1 BM6 fiber condition had a shorter fiber size as compared with XTT BM6, the addition of sodium hypochlorite treatment helped to give the same effect as XTT BM6. The effect of soaking with sodium hypochlorite for 1 day helped to remove the lignin, exposing more hydrogen bonding at the surface of the fiber.

Therefore, the fibers were more intact between each other, as proven from the cross-section image of the handsheet TT1BM6 (Figure 6c). Meanwhile, for unclicked fibers, the XTT BM6 fiber condition showed better wet strength due to longer fiber sizes and more surface bonded area as compared with the TT1 BM6 fiber. However, as the fiber length increased up to 480 μm, both clicked and unclicked handsheets showed a decrement of wet strength, although the clicked handsheet still had better wet strength as compared with the unclicked handsheet. A clear comparison could be seen on the same fiber length, as shown in the cross-section image (Figure 8). More fibers pulled out from unclicked handsheet (Figure 8a) as compared with the clicked handsheet (Figure 8b).

**Figure 8:**
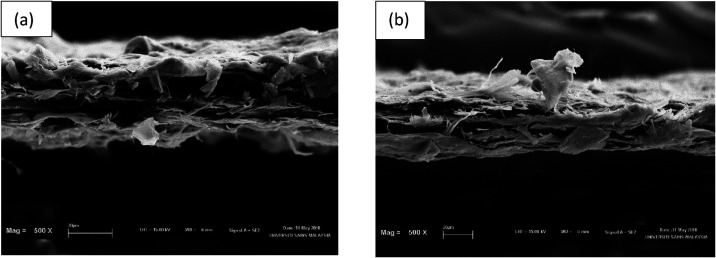
Cross-section SEM images of (a) unclicked XTT XBM and (b) clicked XTT XBM handsheet kapok fibers.

The analysis of variance (ANOVA) of the effect of ball milling and sodium hypochlorite treatment on the wet tensile index of the handsheet (with and without clicking) is shown in Table 1. On the basis of the analysis, both the *F*-value for ball milling (BM) and treatment time (TT) showed a higher value than the *P*-value. The interaction between (BM)*­(TT) factors also showed that the *F*-value was higher than the *P*-value. This indicated that all of the interactions were significant to each other and the wet tensile index of the handsheets.

**Table 1:** ANOVA of the Effect of Ball Milling and Treatment Time on the Wet Tensile Strength of the Handsheets

source	DF	sum of squares	mean squares	*F*-value	*P*-value
ball milling (BM)	3	37.775	12.592	89.396	0.000^a^
treatment time (TT)	3	70.975	23.658	89.396	0.000^a^
(BM)*(TT)	9	26.564	2.952	20.955	0.001^a^

a Statistically significant (*P*-value < 0.05).

### 3.4 Wet Bursting Strength (Bursting Index)

The wet bursting index was measured to observe the strength resistance of the fibers when pressure was applied. Figure 9 shows the bursting index strength for both clicked and unclicked handsheets with different fiber lengths. The clicked handsheet was still superior to the unclicked handsheet as far as the strength was concerned. However, the increase was not too obvious. A similar pattern was also observed as the fiber length was increased for both clicked and unclicked handsheets. Basically, the factors that most affect the bursting properties were the fiber length, fiber formation, and modification or additive addition in the handsheet.^30,31^ The longer the fiber length, the more bonding between the fibers could be formed, leading to an increase in the bursting strength.^32,33^ The flexibility of the fibers also played an important role since longer fibers were more flexible compared with shorter fibers.^34^

**Figure 9:**
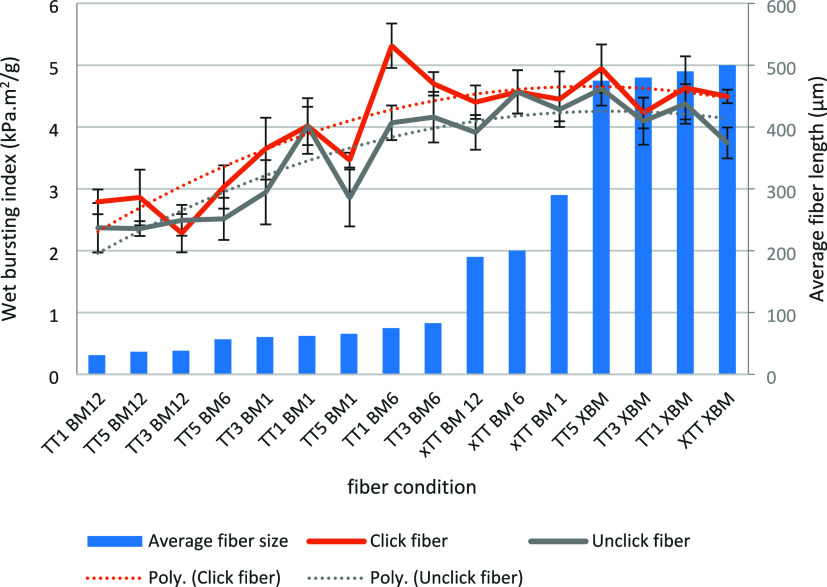
Wet bursting index for clicked and unclicked handsheets with different fiber lengths.

The clicked TT1 BM6 handsheet still possessed the highest wet bursting strength of all. The fibrillation around the fibers helped in providing more sites for modifications and fiber-to-fiber bonding area.

Based on the analysis of variance (ANOVA) of the effect of ball milling and sodium hypochlorite treatment on the wet bursting index of the handsheet (with and without clicking) in Table 2, both the *F*-values for ball milling (BM) and treatment time (TT) showed a higher value than the *P*-value. For the interaction between (BM)*­(TT) factors, the *F*-value was higher than the *P*-value. This indicated that all of the interactions were significant to each other and to the wet bursting index of the handsheet.

**Table 2:** ANOVA of the Effect of Ball Milling and Treatment Time on the Wet Bursting Strength of the Handsheets

source	DF	sum of squares	mean squares	*F*-value	*P*-value
ball milling (BM)	3	8.023	2.674	20.371	0.000^a^
treatment time (TT)	3	69.052	23.017	175.321	0.000^a^
(BM)*(TT)	9	8.254	0.917	6.985	0.002^a^

a Statistically significant (*P*-value < 0.05).

### 3.5 Water Absorption

A water absorption test was conducted to observe the effect of clicking on the handsheets with different fiber sizes. As a potential adsorbent for wastewater treatment, the lower the water absorption, the better. In Figure 10, only selected handsheets were used to compare them with the control handsheet (unclicked XTT XBM).

**Figure 10:**
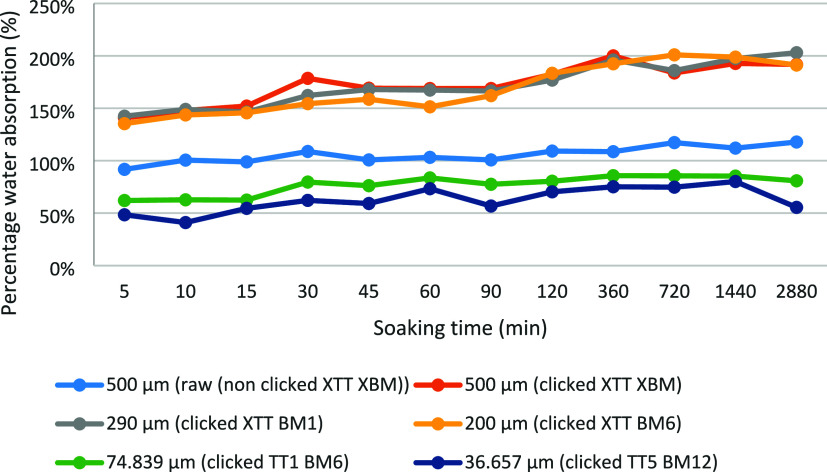
Percentage of water absorption for clicked and unclicked handsheets with different fiber lengths.

Based on the results, it could be seen that the clicked handsheet with smaller fiber size (TT5 BM12) absorbed the lowest percentage of water as compared with the control handsheet (unclicked XTT XBM), followed by the second lowest fiber size, which was the handsheet with clicked TT1 BM6 condition. The effect of treatment and modification seemed to reduce water absorption toward the surface of the clicked handsheet. Based on the average pore size on the surface of the handsheet in Table 3, most of the handsheets were in macropore conditions. The TT5 BM12 handsheet showed the smallest average pore size.

**Table 3:** Average Pore Size for Each Handsheet with Different Conditions

handsheet condition	average fiber length (μm)	average pore size (μm)
control (unclicked XTT XBM)	500	0.5542
clicked XTT XBM	500	0.6607
clicked XTT BM1	290	0.6048
clicked XTT BM6	200	0.5935
clicked TT1 BM6	74.84	0.3253
clicked TT5 BM12	36.66	0.3254

Normally, sodium hypochlorite would smoothen the fiber surface and also create larger pores.^35^ However, longer exposure to sodium hypochlorite would also weaken the fibers.^36,37^ Moreover, ball milling also crushed the fiber into smaller particles. During clicking, a new handsheet was formed, and the distance between the smaller fiber sizes was shorter (Figure 6d). Since the pore size was small, the surface retention and the capillary forces would be affected.^38^ The pore size became much smaller, and the water absorption dropped about 40% from the raw handsheet. Despite that, the rate of water absorption seemed to be maintained as the exposure in water was prolonged.

Different phenomena were observed for unclicked and clicked XTT-XBMs. The clicked XTT XBM showed higher water absorption as compared with the control (unclicked XTT XBM) condition. From the result of the average pore size, the clicked XTT XBM handsheet showed a larger pore size as compared with the unclicked XTT XBM. This might be attributed to the effect of clicking, which held the fiber bonding. During contact with water, the fibers began to swell evenly until they reached their saturation point. For clicked fibers, the clicking had already held the fiber-to-fiber bonding at the surface.^12^ During exposure to water, the swelling effect occurs at the fiber surface that was not bonded. Thus, the gap between the fibers would be much different compared with the unclicked condition. The pore size would be slightly bigger, and more water would easily penetrate into the clicked handsheet (Figure 11). The same phenomenon occurred for both clicked XTT BM1 and clicked XTT BM6 handsheets.

**Figure 11:**
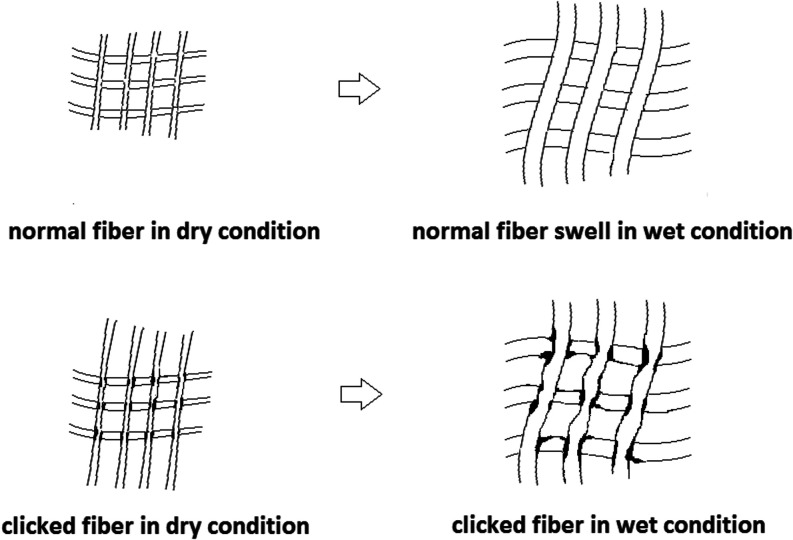
Illustration of the normal and clicked fibers before and after water absorption.

For clicked XTT BM1 and clicked XTT BM6, the pore size was much bigger as compared with the unclicked handsheet due to the fibrillation effect from the ball milling process. From the average pore size result, it could be seen that longer exposure to the ball milling process could also affect the pore size. Clicked XTT BM6 produced a smaller pore size compared with clicked XTT BM1. The fibrillation at the surface of the fiber exposed more microfibers to each other and, at the same time, decreased the pore size at the surface of the fiber (Figure 12). Overall, focusing on wet application, clicked TT1 BM6 showed moderate water absorption. This helped the handsheet to sustain in water for a longer period before disintegration.

**Figure 12:**
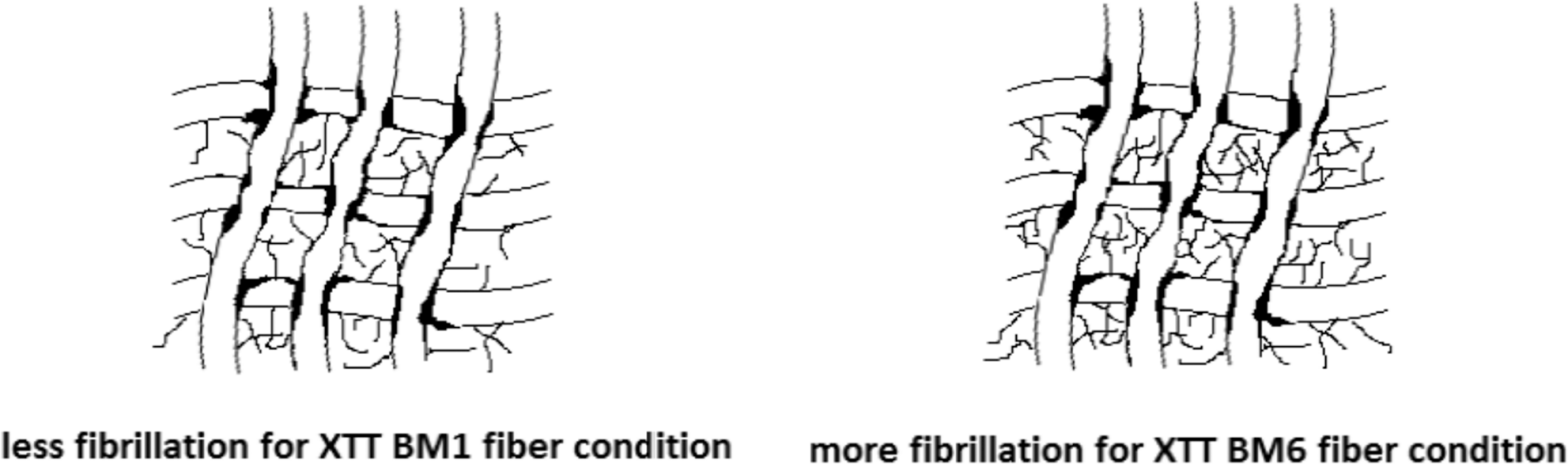
Illustration of the fibrillation condition in handsheets XTT BM1 and XTT BM6.

## 4 Conclusion

The modification of kapok fibers via azide–alkyne click chemistry changed the properties of the fibers under both dry and wet conditions. Even without the presence of copper­(II) as a catalyst, the modification via azide–alkyne click could still be achieved with reduced toxicity, time, and chemical consumption. Moreover, the modification was successfully performed using different fiber sizes. Based on the mechanical property results, the wet tensile index and the wet bursting index decreased with the decrease of fiber size. Prolonged exposure to sodium hypochlorite treatment and ball milling time shorten the fibers, causing less surface available for the cross-linking to take place. The clicked handsheet performed better in both wet tensile and wet bursting properties. In terms of physical properties, the results were varied based on the pore conditions. All handsheets were found to contain macropores. Treatment with sodium hypochlorite and ball milling produced small pores. The longer the treatment and ball milling times, the smaller the pores would be. However, in comparison between the unclicked and clicked handsheets, both unclicked XTT XBM and clicked XTT XBM showed different average pore sizes even though the same average fiber length was used. The effect of clicking made the pore slightly larger as compared with the unclicked condition. Overall, the clicked TT1 BM6 handsheet was the best. It had the highest wet tensile and wet bursting strength with a moderate amount of water absorption among all handsheets.
